# HLA-DR expression in clinical-grade bone marrow-derived multipotent mesenchymal stromal cells: a two-site study

**DOI:** 10.1186/s13287-019-1279-9

**Published:** 2019-06-13

**Authors:** Marta Grau-Vorster, Anita Laitinen, Johanna Nystedt, Joaquim Vives

**Affiliations:** 1 Servei de Teràpia Cel·lular, Banc de Sang i Teixits, Edifici Dr. Frederic Duran i Jordà, Passeig Taulat, 116, 08005 Barcelona, Spain; 2 Transfusion Medicine Group, Vall d’Hebron Research Institute (VHIR), Universitat Autònoma de Barcelona, Passeig de la Vall d’Hebron 129-139, 08035 Barcelona, Spain; 3 Finnish Red Cross Blood Service, Advanced Cell Therapy Centre, Kivihaantie 7, FIN-00310 Helsinki, Finland; 4 Musculoskeletal Tissue Engineering Group, Vall d’Hebron Research Institute (VHIR), Universitat Autònoma de Barcelona, Passeig de la Vall d’Hebron 129-139, 08035 Barcelona, Spain; 5 Departament de Medicina, Universitat Autònoma de Barcelona, Passeig de la Vall d’Hebron 129-139, 08035 Barcelona, Spain

**Keywords:** Multipotent mesenchymal stromal cell, Potency, Identity, HLA-DR, Cellular therapy, Cell culture, Quality compliance

## Abstract

**Background:**

Contrary to the minimal criteria proposed by the International Society for Cell and Gene Therapy for defining multipotent mesenchymal stromal cells (MSC), human leukocyte antigen (HLA)-DR expression is largely unpredictable in ex vivo-expanded clinical-grade cultures. Although activation of MSC in culture does not appear to affect their functionality, a large study investigating the impact of HLA-DR expression on cell identity and potency is still missing in the literature.

**Methods:**

A retrospective analysis of HLA-DR expression in 130 clinical batches of bone marrow (BM)-MSC from two independent Good Manufacturing Practice-compliant production facilities was performed in order to identify the consequences on critical quality attributes as well as potential activation cues and dynamics of MSC activation in culture.

**Results:**

HLA-DR^+^ cells in culture were confirmed to maintain fibroblastic morphology, mesenchymal phenotype identity, multipotency in vitro, and immunomodulatory capacity. Interestingly, the use of either human sera or platelet lysate supplements resulted in similar results.

**Conclusions:**

HLA-DR expression should be considered informative rather than as a criterion to define MSC. Further work is still required to understand the impact of HLA-DR expression in the context of product specifications on BM-MSC qualities for clinical use in specific indications.

**Electronic supplementary material:**

The online version of this article (10.1186/s13287-019-1279-9) contains supplementary material, which is available to authorized users.

## Introduction

Multipotent mesenchymal stromal cells (MSCs) can be easily derived from bone marrow (BM) aspirates and further expanded ex vivo up to sufficient numbers for clinical use [1, 2]. Indeed, several clinical applications of MSC are currently being explored in the fields of transplantation and regenerative medicine, with a couple of products already holding marketing approval [3–5]. In addition to their potential to become specialized bone, cartilage, and fat cells, the use of MSC is of particular interest in the management of graft rejection and/or inflammatory conditions, due to their capacity to modulate the biological response of other cell types involved in these processes [6]. In view of their increasing popularity of MSC as a candidate drug for use in the treatment of a range of pathologies, the International Society for Cell and Gene Therapy (ISCT) established minimal criteria to define MSC identity in an attempt to standardize the product specifications and methods for characterization of MSC regardless of their origin and derivation protocols [7]. ISCT’s criteria are based on MSC’s adherence to plastic surfaces, multipotency in vitro, and the expression of a set of cell surface markers (positive for CD73, CD90, and CD105; negative for CD14, CD34, CD45 or CD11b, CD79α or CD19, and HLA-DR). Intriguingly, BM-MSC do express HLA-DR not only when inflammatory molecules such as IFN-γ are present but also under normal expansion culture conditions [8, 9].

In the present study, we re-analyzed HLA-DR expression in 130 clinical batches of BM-MSC from two independent Good Manufacturing Practice (GMP)-compliant production facilities to study its potential effect on the attributes of MSC and the dynamics of activation in expansion cultures.

## Methods

### Cells and cell culture

In Barcelona, clinical-grade BM-MSC were produced within the context of four clinical trials (2009-016449-24, 2010-023998-18, 2013-005025-23, 2010-023999-12) with appropriate donor informed consent and approval from a competent regulatory authority. When needed, cells were further expanded in vitro up to sufficient numbers (always under passage 4) using Dulbecco’s modified Eagle’s medium (DMEM; Gibco) supplemented with 10% human serum B (hSerB) containing 2 mM glutamine in T-flasks and CellSTACKS (Corning Incorporated Life Sciences) at 1 × 10^3^ to 3.5 × 10^3^ cells/cm^2^ seeding density [10]. All cultures were maintained at 37 °C and 5% CO_2_ in humidified incubators. Cell counting and viability assessment of BM samples were performed using a Guava EasyCyte Mini (Millipore).

In Helsinki, donor recruitment, eligibility assessment, and bone marrow aspiration protocol were approved by the local ethics committee, and the BM collection procedure was authorized by the Finnish Medicines Agency under a tissue establishment license. MSCs were produced under a national advanced therapy medicinal products (ATMP) hospital exemption license authorized by the Finnish Medicines Agency (Fimea; national ATMP manufacturing licenses #6322/20.10.01/2011 and #5103/20.30.01/2013). MSC were derived from BM aspirates as reported elsewhere [11]. Briefly, primary cultures (P0) were initiated by plating isolated BM mononuclear cells (MNCs) at 4 × 10^5^ cells/cm^2^. The MSCs were expanded in a medium consisting of Dulbecco’s modified Eagle’s medium (DMEM) low glucose (Life Technologies) supplemented with 10% human Platelet Lysate (hPL) and 40 UI/mL heparin (Heparin LEO 5000 IE/KY/mL; Leo Pharma) [12]. Culture medium was replaced twice a week, and the cells were detached with TrypLE Select CTS (Life Technologies). The cells were plated at a density of 1 × 10^3^ cells/cm^2^ from P1 onwards.

### Phenotype assessment

Immunophenotypic characterization of BM-MSC was performed using the following antibodies. The Barcelona protocol used mouse anti-human CD45-fluorescein isothiocyanate (CD45-FITC, HI30, BD Pharmingen), anti-human CD105-phycoerythrin (CD105-PE, 43A4E1; Miltenyi Biotec), anti-human HLA-DR-FITC (L243; BD Biosciences), anti-human CD90-PE (F15-42-1-5, Beckman Coulter), mouse anti-human CD31-FITC (WM59; BD Pharmingen), and mouse anti-human CD73-PE (AD2, BD Pharmingen). The Helsinki protocol used cell surface antigens CD44 (CD44 –FITC, G44-26; BD Pharmingen), CD49e (CD49e-PE, IIA1; BD Pharmingen), CD13 (CD13-APC, WM15; BD Pharmingen), CD90 (CD90-FITC, 5E10; Stemcell Technologies and BD Pharmingen), CD73 (CD73-PE, AD2; BD Pharmingen), CD29 (CD29-APC, MAR4; BD Pharmingen), CD105 (CD105-PE, 266, BD Pharmingen; 43A3, Stem Cell Technologies; and MEM-229, Abcam), CD14, (CD14-PE, M5E2; BD Pharmingen), CD19 (CD19-PE, HIB19; BD Pharmingen), CD34 (CD34-PE, 8G12, BD Biosciences; AC136, Miltenyi Biotec), CD45 (CD45-PE, H130; BD Pharmingen), and HLA-DR (HLA-DR –PE, MEM-12, Abcam; L243, BD Biosciences).

Cells were stained for 15 min at room temperature (Barcelona) and for 30 min on ice (Helsinki), washed, and resuspended in phosphate-buffered saline (DPBS; Gibco). Non-specific cell staining was ruled out by using mouse immunoglobulin isotype controls (BD Pharmingen). The acquisition was performed on a FACSCalibur (Barcelona) or a Navios cytometer (Helsinki), and data were analyzed with the FlowJo software (TreeStar Inc., Ashland, OR, USA).

In both laboratories, HLA-DR expression was analyzed and used only for informative purposes but not for product release.

Re-analysis of HLA-DR for the standardization of data analysis from the two groups was performed using FlowJo. The gating strategy with the use of IgG isotype control for unspecific staining (below 1%) is shown in Additional file 1: Figure S1.

### Differentiation assays

Specific differentiation media were used for the osteogenic, chondrogenic, and adipogenic induction of undifferentiated MSC cultures in vitro as reported previously [12–14]. Safranin O (Sigma), Oil Red O (Sigma), Sudan III (Sigma), and von Kossa (Sigma) stainings were performed for the determination of the outcome of the differentiation assays.

### Cell count, viability, and apoptosis

Cells were counted either by Trypan blue dye exclusion methods or with Perfect-Count Microspheres (Cytognos) in a FACSCalibur cytometer (Becton Dickinson). Viability was determined using the 7-amino-actinomycin D (7-AAD, BD Biosciences) exclusion method and expressed as a percentage (%) of total cells. Data were analyzed with the CellQuest Pro (Becton Dickinson) software. Alternatively, cell numbers and viability were determined using the NucleoCounter NC-100 device (ChemoMetec A/S, Denmark).

### Lymphocyte proliferation assays

The immunomodulation potential of BM-MSC was tested in the presence of peripheral blood mononuclear cells (PBMC) at a ratio of 1:5 (Barcelona procedure) and 1:10, 1:20, and 1:50 (Helsinki procedure) as described elsewhere [12, 15]. The proliferation of carboxyfluorescein diacetate succinimidyl ester (CFSE)-labeled PBMC after polyclonal stimulation was determined by measuring the reduction of fluorescence intensity at day 5 by flow cytometry.

### Effect of different medium supplements

The effect of different medium supplements on the variation of HLA-DR expression was investigated after incubation of the same BM-MSC lines with different batches of sera. For this purpose, three independent BM-MSC lines were expanded under the same conditions for one passage and subsequently incubated with either one of two different conditions for an additional passage: one condition that was previously found experimentally related to activation of BM-MSC (14.3 ± 12.8% HLA-DR^+^ cells, *n* = 7) and another one related to low HLA-DR^+^ cell number in culture (3.3 ± 3.3% HLA-DR^+^ cells, *n* = 2).

### Activation of MSC

For cell activation experiments, IFN-γ (R&D Systems, Minneapolis, MN, USA) was used as a stimulus at 10, 100, and 200 UI/mL, based on the concentrations used in previously reported experiments [16].

For the recovery after stimulation experiments, the same line was used for all three conditions. Cells were previously stimulated 48 h with 100 UI/mL of IFN-γ. After 48 h incubation, cells were left without IFN-γ to see the decrease in HLA-DR. Controls without stimulation at any point and with maintained stimulation with 100 UI/mL of IFN-γ were performed.

### Data analysis

Descriptive data were expressed as mean ± standard deviation. Pearson correlation *p* values were calculated to evaluate the significance of the correlation of cell density and HLA-DR expression in BM-MSC.

## Results

### BM-MSC express variable levels of HLA-DR

**Fig. 1:**
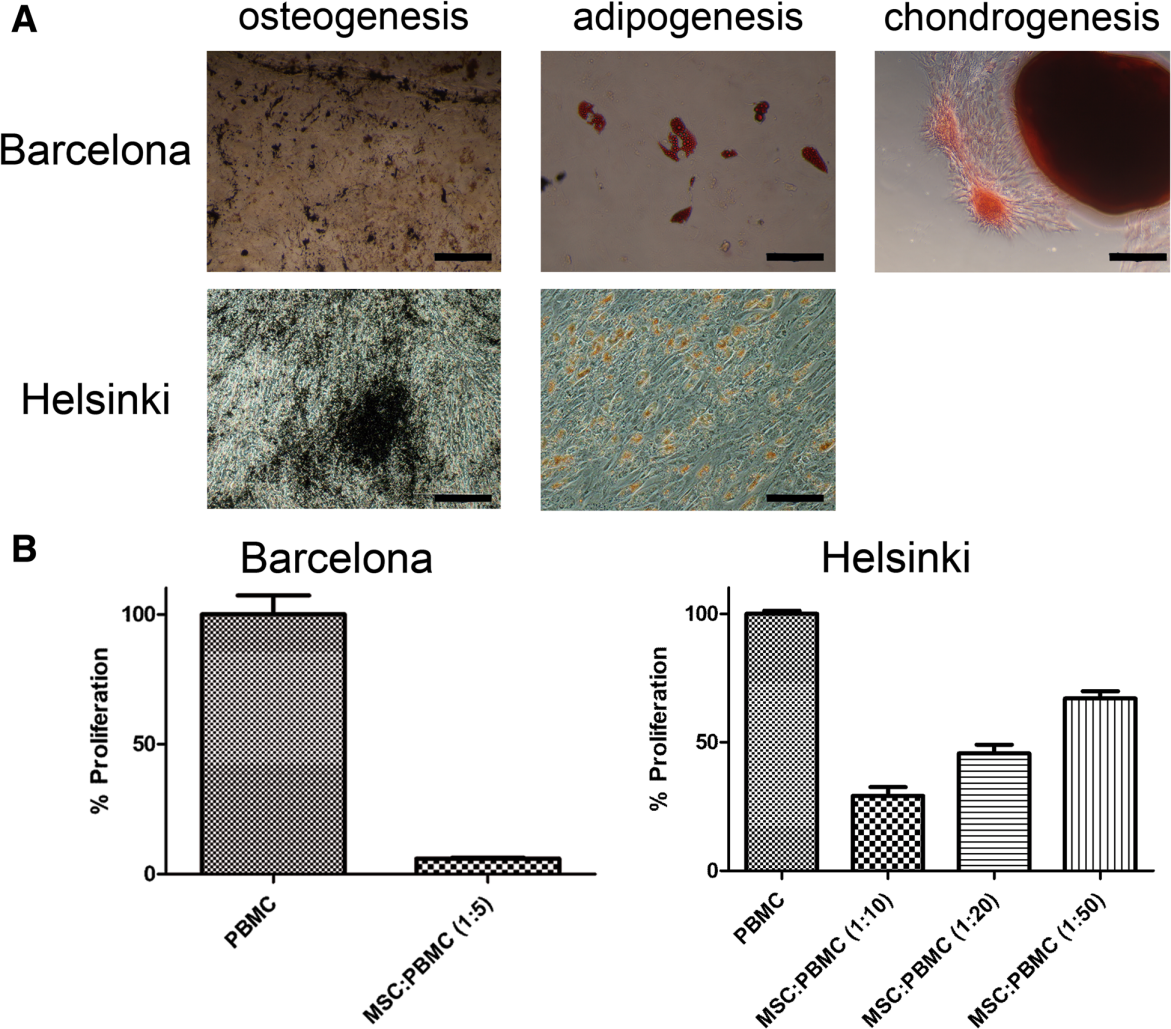
Differentiation and immunomodulation potential of BM-MSC. Selected examples of characterization assays performed on BM-MSC at Barcelona’s and Helsinki’s facilities. **a** Differentiation potential was confirmed by von Kossa staining after 4-week culture in osteogenic medium (black depositions), Oil Red O/Sudan III staining after 3-week culture in adipogenic medium (lipid droplets in the cytoplasm of adipocytes stained red), and Safranin O staining after 3-week culture in chondrogenic medium (glycosaminoglycans stained red). **b** Immunomodulatory properties of MSC were tested by means of the lymphocyte proliferation assay, in which MSC demonstrated their capacity to suppress proliferation of polyclonally stimulated lymphocytes. Barcelona scale bars = 200 μm. Helsinki von Kossa scale bars = 500 μm and Oil Red O scale bars = 100 μm

Despite compliance with the ISCT criteria, BM-MSC in culture showed variable percentages of HLA-DR^+^ cells in 130 batches produced in two manufacturing sites (namely, Barcelona, *n* = 91, and Helsinki, *n* = 39). HLA-DR expression in Barcelona batches ranged from < 1 to 77.7% (average 19.8 ± 15.6%, from patients enrolled in clinical trials for autologous use), whereas batches in Helsinki ranged from < 1 to 60.5% (average 19.2 ± 17.4%, from healthy donors for allogeneic use) (Table 1). In order to bring consistency to the comparability of analyses performed in different laboratories following different techniques, we reanalyzed a fraction of data from both laboratories (*n* = 65 and *n* = 37 from Barcelona and Helsinki, respectively; Additional file 1: Figure S2) BM-MSC batches from both centers complied with the specifications approved by their own respective regulatory authority in terms of phenotype, adherence to culture-treated plastic surfaces, fibroblastic morphology, differentiation potential, and immunomodulation capacity in vitro (Additional file 1: Table S1, Fig. 1).

**Table 1:** Summary of HLA-DR expression in 130 batches of clinical-grade BM-MSC from two independent GMP-compliant facilities. Batches of BM-MSC were distributed according to the production site

Group	Number	Mean (%)	Standard deviation (%)	Minimum (%)	Maximum (%)
Barcelona	91	19.8	15.6	< 1	77.7
Helsinki	39	19.2	17.4	< 1	60.5
Total	130

In order to determine the potential triggers of cell activation in culture, standard parameters were analyzed, including density, medium supplements, and forced activation with IFN-γ and its recovery after removal of the stimulus.

### Effect of cell density and passaging on HLA-DR expression

**Fig. 2:**
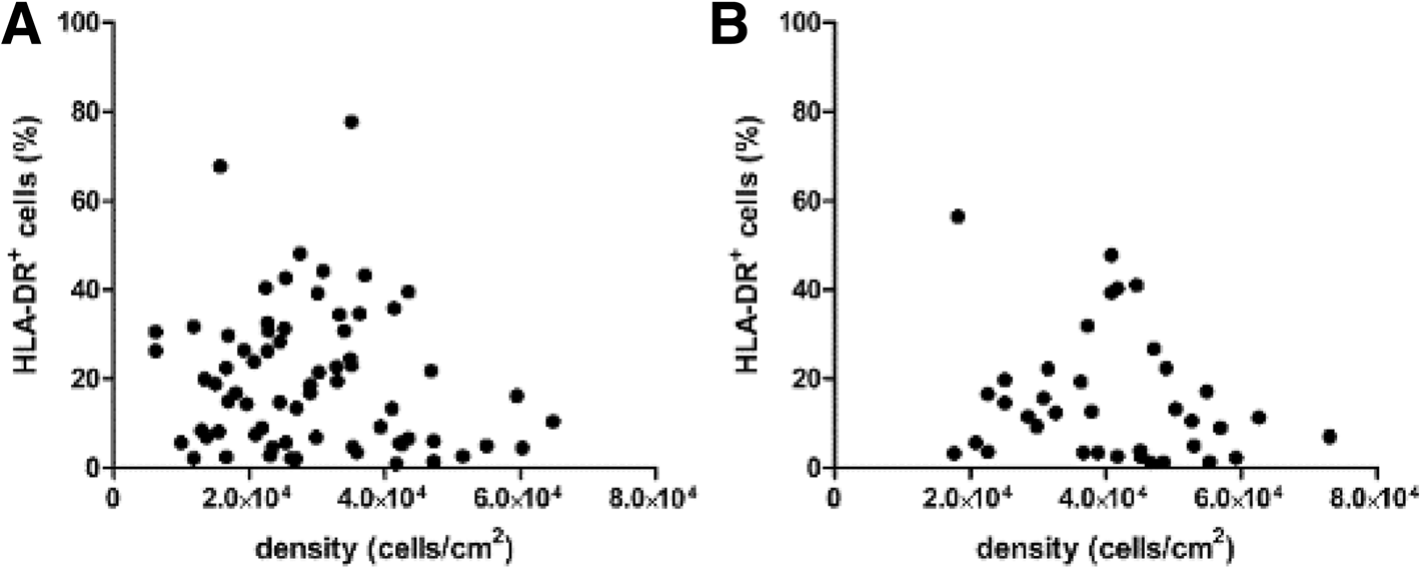
Analysis of cell culture parameters. Effect of cell density on HLA-DR expression was studied in batches released from both manufacturing sites, namely Barcelona (**a**) and Helsinki (**b**). However, high dispersion without statistical correlation was observed in both cases (*p* value = 0.2287 and 0.2272, respectively)

Regarding cell density and HLA-DR expression, no correlation was found (Fig. 2); dispersion without statistical correlation was observed in both cases (*p* value = 0.2287 and 0.2272, in Barcelona and Helsinki datasets, respectively). However, culture passaging was found to change the HLA-DR^+^ cell percentage. We collected HLA-DR values from the two passages required in the production process, which typically take a total of 3 weeks and correspond to 24.6 ± 1.5 cumulative population doublings (CPD). We observed a decrement in HLA-DR positivity of at least 25% in 71.9% of the cultures, whereas only 5.3% increased HLA-DR more than 25% of its initial value (*n* = 57). Furthermore, one MSC line was cultured up to passage 5 confirming the decrease in HLA-DR^+^ cell number (Additional file 1: Figure S3).

### Effect of cell culture medium supplement on HLA-DR expression

Different batches of sera experimentally related with high and low HLA-DR values were tested in three BM-MSC lines derived from three different donors. Despite starting with similar HLA-DR values (17.1%, 19.4%, and 15.0%; corresponding to an average of 17.2% ± 2.2%), the percentage of activation after incubation varied independently of the cell line or supplement. Remarkably, none of the other mesenchymal surface markers depended on HLA-DR expression (Table 2). Additionally, the potentiality of the three cell lines was not altered in either case, showing a similar capacity to differentiate in vitro into the osteogenic and chondrogenic lineages (Additional file 1: Figure S4).

**Table 2:** Result summary after expanding cells in different medium formulations associated with “activating” or “neutral” cues. Activation of cells occurred randomly regardless of the supplement used. Nonetheless, differentiation potential was not altered (please refer to Additional file 1: Figure S2)

Cell line	Passage number	supplement	%CD31	%CD45	%HLA-DR	%CD73	%CD105	%CD90
1	P0		3.6	1.9	*17.1*	99.8	99.9	99.83
	P1	Activating	2.0	0.3	*14.2*	99.7	99.9	99.6
	P1	Neutral	7.7	2.0	*29.6*	99.3	99.4	99.4
2	P0		6.4	1.1	*19.4*	100	98.7	99.9
	P1	Activating	8.4	3.0	*37.7*	99.7	99.6	99.9
	P1	Neutral	7.6	3.3	*40.9*	99.8	99.5	99.9
3	P0		7.2	1.0	*15.0*	99.7	99.7	99.84
	P1	Activating	4.7	3.3	*23.9*	99.4	99.4	99.5
	P1	Neutral	4.3	2.9	*25.4*	99.1	99.3	99.3

*P* passage number

### Activation of HLA-DR expression

**Fig. 3:**
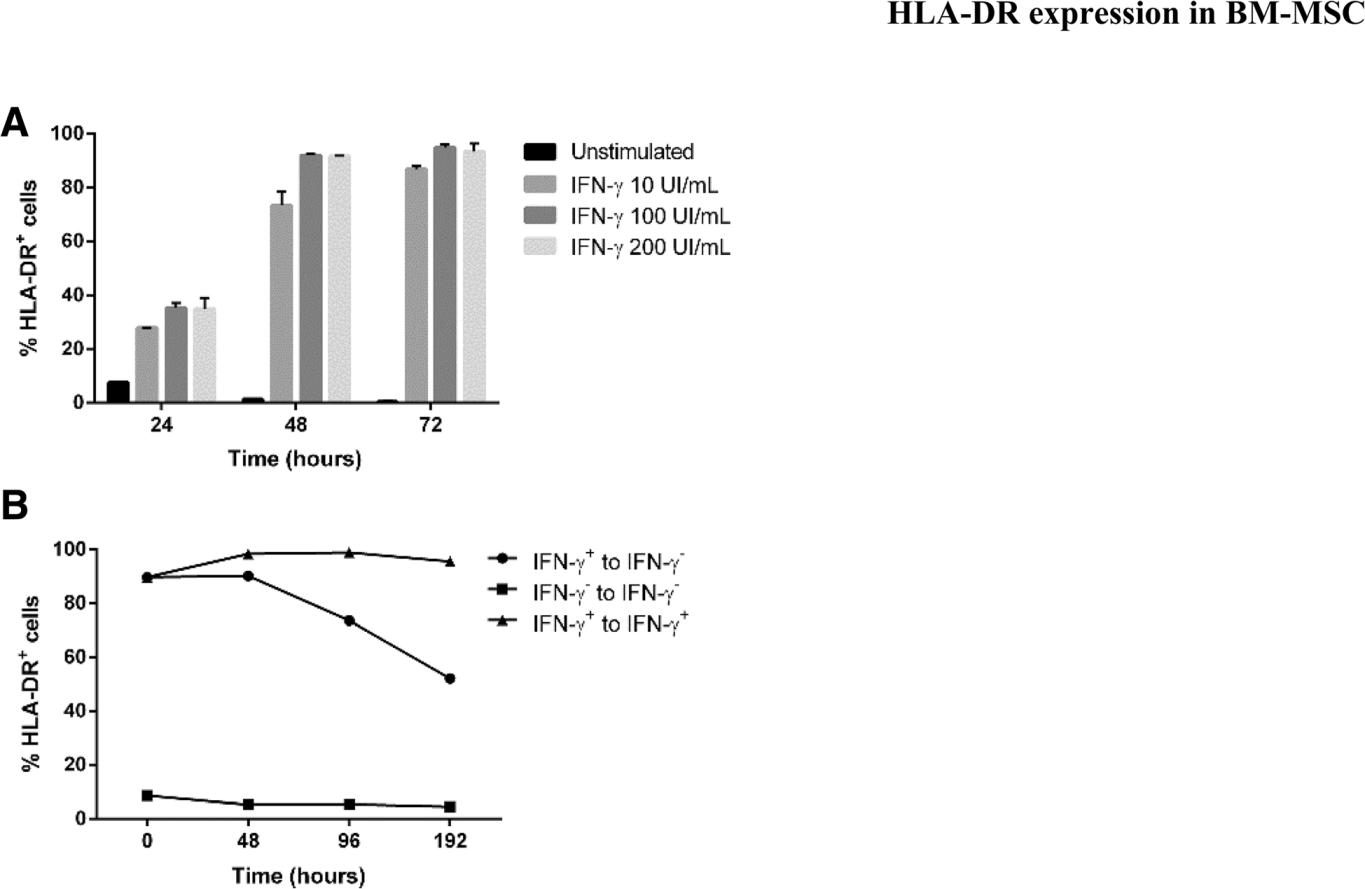
Dynamics of BM-MSC activation in vitro. In culture, BM-MSC are activated by the addition of IFN-γ, which is illustrated by increasing concentration up to 200 UI/mL, reaching a peak 48 h after stimulation (**a**). Interestingly, the reversal of the process for the recovery of HLA-DR expression levels using IFN-γ-activated cells takes longer than the activation time (**b**)

We took advantage of IFN-γ being a strong activator of MSC in vitro and performed a series of experiments to study the effect of its addition to determine the kinetics of BM-MSC activation upon stimulation with increasing doses of IFN-γ. We observed that the maximum activation was reached at 48 h after induction, resulting in high levels of activation when using 100 and 200 UI/mL doses (Fig. 3a). Interestingly, when IFN-γ stimulus was removed, the number of HLA-DR^+^ cells decreased gradually over time (Fig. 3b).

## Discussion

Variability of HLA-DR expression in MSC cultures is a nuisance for developers adhering to the criteria established by the ISCT, so it tends to be overlooked provided that quality control (QC) panels are adapted to robust product specifications in order to bring consistency from batch to batch using reliable markers [17]. This makes it difficult to fully realize how different cell preparations from different production sites can be. In this context, standardization of QC criteria and methodology is a pressing need as new MSC-based therapies reach the clinical setting and comparability of studies is required to define the efficacy of such new drug candidates [4, 5].

In the present study, we focused on the analysis of HLA class II molecule expression in cultured MSC which was demonstrated to be unpredictable and dynamic over time thus confirming previous studies [8]. Expression of HLA-DR has been proved, both in the present work and reported by others, that it can be triggered in vitro by IFN-γ [9, 18] and other pro-inflammatory stimuli [19]. Interestingly, when IFN-γ stimulus was removed, the number of HLA-DR^+^ cells gradually decreased over time. This observation is relevant since HLA-DR expression on cells during expansion cultures is variable over time; however, in-process and final product quality controls are performed only at specific time points during the production process, typically after 8 to 10 days from seeding, therefore showing only a snapshot of the status of the cells and as a result could be potentially masking a dynamic pattern of expression of HLA-DR. Regardless of HLA-DR, the identity of MSC is clearly defined by other parameters, such as the presence of mesenchymal cell surface markers and absence of hematopoietic and endothelial markers, fibroblastic cell morphology on plastic surfaces, and immunomodulation and differentiation potential [7]. Indeed, we also showed that HLA-DR expression had no effect on immunomodulation and multilineage differentiation potential of MSC [8].

In one of the first reports on this topic, Le Blanc and collaborators analyzed the intracellular protein levels of HLA-DR in MSC in parallel with extracellular HLA-DR phenotype by flow cytometry [18]. They found that MSC expressed first HLA-DR at the protein level intracellularly, and only later HLA-DR molecules were detected in the cellular membrane upon activation. The intracellular synthesis of HLA-DR was initiated after 2 days of exposure to IFN-γ, but required up to 7 days for cell surface expression. Nevertheless, we detected MSC activation extracellularly after 48 h after IFN-γ exposure and provided data of slow HLA-DR downregulation. With this, it seems clear from these studies that the expression of HLA-DR can come from a previous exposure since clinical-grade manufacture bioprocesses are relatively short (that is, two passages only) and we provided evidence of variability of HLA-DR expression even in the very first passage. One might think that the condition of the donor could determine MSC activation in culture; however, our study collected data from both healthy donors and patients, and in both cases, the occurrence of HLA-DR expression appeared to be random.

With the removal of HLA-DR expression of MSC from the QC panels, and the acceptance that HLA-DR expression in cultured MSC does not follow a predictable pattern under standard in vitro growth conditions, it would be interesting to further investigate its triggers as its expression is known to be indicative of inflammatory microenvironments and maybe also differentiation commitment [20]. In any case, the major concern regarding HLA-DR expression in MSC would be the potential rejection of these cells. Lately, new hypotheses about the MSC mechanisms of action involved are being proposed, and among them, efferocytosis is gaining strength. This hypothesis supports that MSC releases soluble molecules and extracellular macrovesicles that signal to the target environment and then disappear [6]. Without considering HLA-DR expression, MSCs have not been reported to cause rejection, but on the contrary, MSCs are being used in the management of graft-versus-host disease (GvHD) [21, 22]. The mechanisms by which these cells induce tolerance were reported by Galleu and collaborators in vivo in an animal model and were apoptosis of MSC resulted in the induction of immunosuppression [23]. Interestingly, IFN-γ-activated MSCs are being used for the treatment of GvHD to induce tolerance [9]. Moreover, Zachar and collaborators illustrated that MSCs have to be activated in order to provide their therapeutic effects in the regenerative medicine field [19]. Therefore, the presence of HLA-DR molecules on the cell surface does not compromise MSC identity, neither their function and application in the clinical setting for immunomodulation and differentiation potential applications. Further clinical studies could discard the secondary effects of activated MSC as well as their therapeutic effect.

## Conclusions

We concluded that the use of HLA-DR as a negative marker of BM-MSC does not add any additional value to QC panels and its expression does not affect other attributes, such as phenotype and functionality in vitro. Furthermore, we showed for the first time that the expression of HLA-DR is dynamic in MSC cultures. Finally, we believe that we can only reliably describe the full clinical potential of MSC if robust QC panels directly related to MSC’s functionality are characterized, so the definition and broad adoption of meaningful potency assays are urgently needed.

## Additional file

Additional file 1: **Table S1.** Release criteria of clinical-grade batches of BM-MSC from two independent GMP-compliant facilities. Release criteria defined on MSC phenotype. **Figure S1.** Gating strategy used in flow cytometry analysis to determine HLA-DR expression in BM-MSC. Represented in blue IgG isotype control for unspecific staining. The interval gate was established with a value of HLA-DR^+^ of isotype control positivity below 1%. **Figure S2.** Comparison of HLA-DR covariance between batch release and after standardization of the analysis of cytometric data from Barcelona and Helsinki. Statistically not significant (*p* value 0.2997). **Figure S3.** Percentage of MSC expressing HLA-DR along culture passaging. **Figure S4.** Osteogenic and chondrogenic potential of cells cultured in the presence of activating and non-activating sera supplements. Scale bars = 200 μm. (DOCX 16212 kb)

## Data Availability

All datasets generated for this study are included in the manuscript and the supplementary files.

## Abbreviations

BM: Bone marrow
CFSE: Carboxyfluorescein diacetate succinimidyl ester
CPD: Cumulative population doublings
FITC: Fluorescein isothiocyanate
GMP: Good Manufacturing Practice
HLA: Human leukocyte antigen
MSC: Multipotent mesenchymal stromal cells
P: Passage number
PBMC: Peripheral blood mononuclear cells
PE: Phycoerythrin
QC: Quality control
