# Supplementary material for: HLA-DR expression in clinical-grade bone marrow-derived multipotent mesenchymal stromal cells: a two-site study
Source: Stem Cell Res Ther. 2019 Jun 13;10:164. doi: 10.1186/s13287-019-1279-9 (PMC6567533; doi:10.1186/s13287-019-1279-9)

**Additional file 1**

**TABLE S1. Release criteria of clinical grade batches of BM-MSC from two independent GMP-compliant facilities.** Release criteria defined on MSC phenotype.

|  | **Positive** | **Negative** |
| --- | --- | --- |
| **Barcelona** | CD105, CD73, CD90 | CD45, CD31 |
| **Helsinki** | CD44, CD49e, CD13, CD90,  CD73, CD29, CD105 | CD14, CD19, CD34, CD45 |


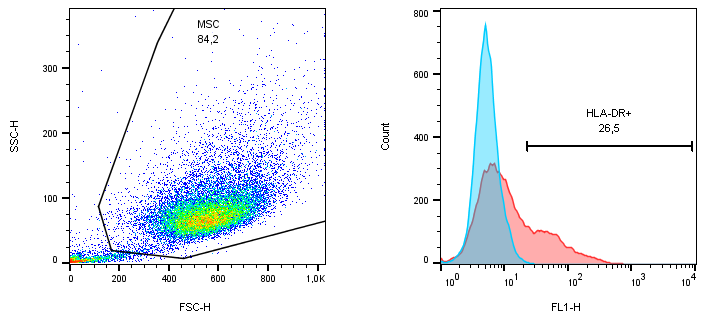


**FIGURE S1.** Gating strategy used in flow cytometry analysis to determine HLA-DR expression in BM-MSC. Represented in blue IgG isotype control for unspecific staining. The interval gate was established with a value of HLA-DR^+^ of isotype control positivity below 1%.

**FIGURE S2.** Comparison of HLA-DR covariance between batch release and after standardization of the analysis of cytometric data from Barcelona and Helsinki. Statistically not significant (p-value: 0.2997).


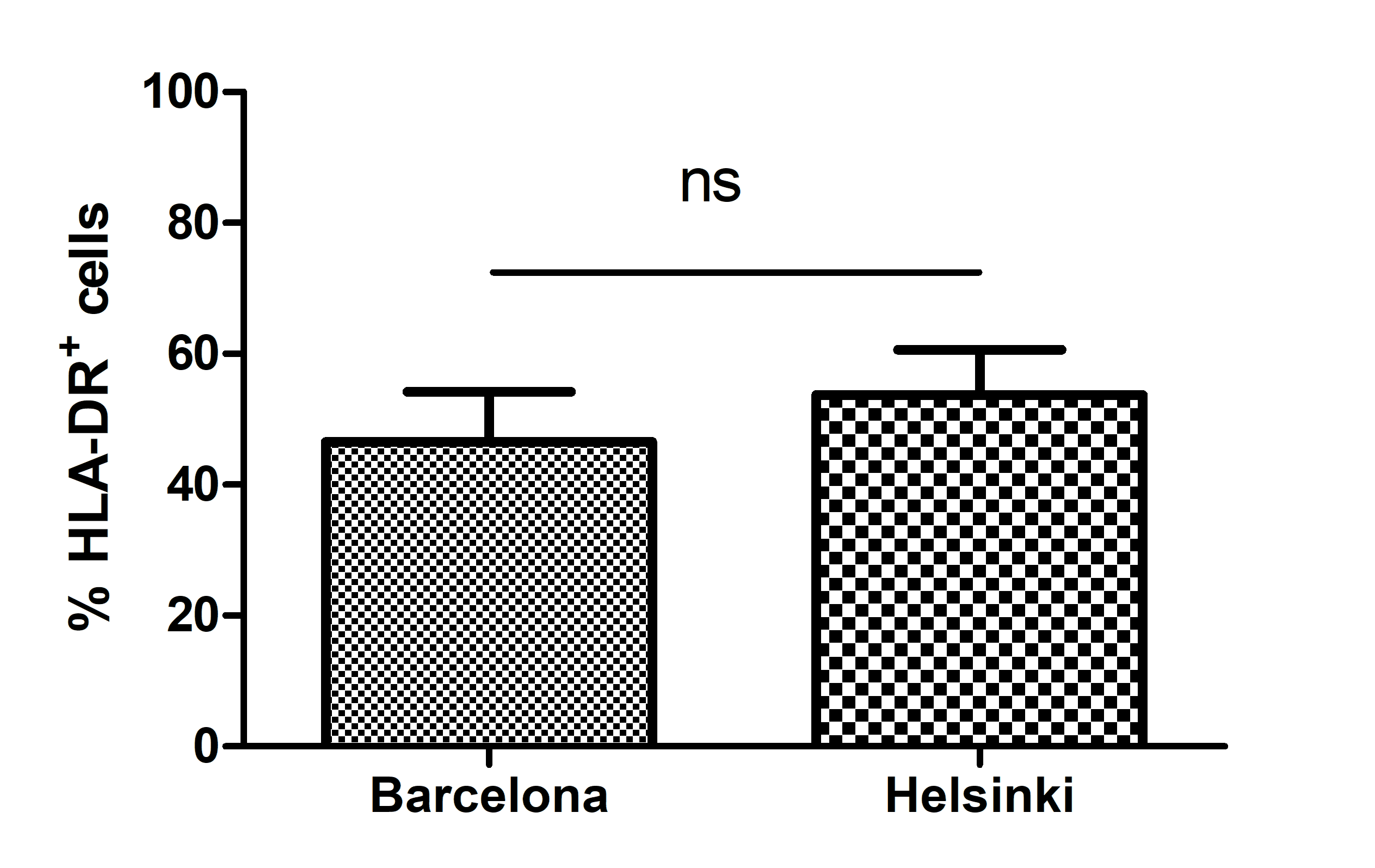


**FIGURE S3.** Percentage of MSC expressing HLA-DR along culture passaging.

**FIGURE S4.** Osteogenic and chondrogenic potential of cells cultured in the presence of activating and non-activating sera supplements. Scale bars = 200 µm.


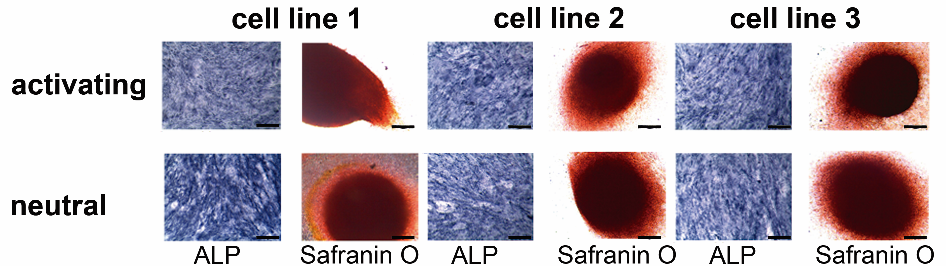

Supplement: Supplementary file 1 — Table S1. Release criteria of clinical-grade batches of BM-MSC from two independent GMP-compliant facilities. Release criteria defined on MSC phenotype. Figure S1. Gating strategy used in flow cytometry analysis to determine HLA-DR expression in BM-MSC. Represented in blue IgG isotype control for unspecific staining. The interval gate was established with a value of HLA-DR+ of isotype control positivity below 1%. Figure S2. Comparison of HLA-DR covariance between batch release and after standardization of the analysis of cytometric data from Barcelona and Helsinki. Statistically not significant (p value 0.2997). Figure S3. Percentage of MSC expressing HLA-DR along culture passaging. Figure S4. Osteogenic and chondrogenic potential of cells cultured in the presence of activating and non-activating sera supplements. Scale bars = 200 μm. (DOCX 16212 kb) [file 13287_2019_1279_MOESM1_ESM.docx]
